# Evaluation of the Health Status of Largemouth Bass (*Micropterus salmoides*) at Different Stocking Densities Under the “168” Aquaculture Model Based on an Integrated Analysis of Liver Histology, Biochemistry, Transcriptomics, and Metabolomics Data

**DOI:** 10.3390/ani16132099

**Published:** 2026-07-07

**Authors:** Meng Yuan, Jianfang Guo, Yifei Sun, Zhihao Liu, Yibo Zhao, Yikai Li, Yongtao Tang, Tianxi Fu, Chuanjiang Zhou

**Affiliations:** 1 College of Life Sciences, Henan Normal University, No. 46, Jianshe East Road, Muye District, Xinxiang 453007, China; 18888526797@163.com (M.Y.); 19712773098@163.com (J.G.); syf581031@gmail.com (Y.S.); 13849013501@163.com (Z.L.); 13007527278@163.com (Y.Z.); liyikai119@163.com (Y.L.); 2 College of Fisheries, Henan Normal University, Xinxiang 453007, China; xiaomitang@126.com; 3 Zhejiang Xinxin Tianen Aquatic Feed Co., Ltd., Jiaxing 314006, China; futianxi2004@126.com

**Keywords:** *Micropterus salmoides*, “168” aquaculture model, stocking density, Ser metabolism, oxidative stress

## Abstract

Largemouth bass (*Micropterus salmoides*) is a major aquaculture species in China. Facility-based aquaculture, such as the “168” model, a high-efficiency recirculating system using funnel-shaped ponds, has promoted water conservation and improved aquaculture efficiency through structural innovation. However, fish die sporadically as the stocking density increases with increasing fish growth. To address this issue, three density groups were established, namely, low (2.5 ± 0.5 kg/m^3^), medium (4.0 ± 0.5 kg/m^3^), and high (7.5 ± 0.5 kg/m^3^). Histological examinations, biochemical assays, and transcriptomic and metabolomic analyses of liver tissues were performed, and fish health was comprehensively evaluated. Histopathological analysis revealed that progressive hepatic vacuolization and severe tissue damage occurred as the fish density increased. Biochemical indicators revealed that the immune system and growth underwent compensatory activation at medium density, shifting to immune suppression, growth impairment, and hepatic exhaustion at high density. Integrated omics analysis revealed that under medium-density stress, the urea cycle was impaired; under high-density stress, Ser metabolism in the liver was rerouted, potentially to overcome methyl donor depletion and prevent disorders of polyamine metabolism, accompanied by a gradual transition from compensatory activation to functional exhaustion. These findings improve our understanding of the physiological response mechanisms of fish to high-density stress. This study provides a theoretical basis for optimizing high-density aquaculture technologies such as the “168” model.

## Simple Summary

Largemouth bass is a highly important farmed fish in China. While the eco-friendly “168” pond system is great for farming them, fish farmers sometimes see unexpected fish deaths. We suspect that this is caused by keeping too many fish in one space, which causes stress and illness. We studied how different crowding levels affect the liver, blood, and internal biology of fish. We found that at low to medium densities, the fish adapt well and remain healthy. However, at high densities, fish suffer from severe stress, weakened immunity, and severe liver damage, increasing their risk of death. To ensure both good production and good fish welfare, we recommend keeping fish at moderate densities, separating them as they grow, and providing good nutrition. This study helps fish farmers raise healthier fish in a more sustainable way.

## 1. Introduction

Largemouth bass (*Micropterus salmoides*) is an economically important freshwater fish in China. In 2024, its annual aquaculture production exceeded 940,000 tonnes [1]. The Food and Agriculture Organization (FAO) has prioritized the largemouth bass as a high-quality freshwater fish for global promotion [2]. This species is among the most industrialized Perciformes fish in global aquaculture, with China serving as the largest producer worldwide. China’s aquaculture sector currently includes a diverse range of models, mainly including traditional pond farming, aquaponics, floating aquaculture vessels, and land-based container aquaculture [1,3]. Although traditional pond aquaculture has mature technology and low entry barriers, it generally faces bottlenecks such as high consumption of water and land resources, high effluent discharge loads, and limited environmental control capacity. While the aquaponics model can achieve a closed-loop nitrogen and phosphorus cycle, it requires complex regulation of the ecological balance of the system and faces limitations in terms of large-scale expansion and species suitability [4]. While floating aquaculture vessels use deep-sea environments to overcome capacity limitations in nearshore areas, they are constrained by high construction and operational costs as well as limited adaptability to sea conditions [5]; land-based container aquaculture provides the advantages of precise environmental control and land use efficiency, but its high energy consumption and investment costs mean that it is largely restricted to high-value-added species [5]. Overall, while existing models each have their own advantages in resource conservation or environmental friendliness, there remains room for optimization in balancing low construction costs, strong environmental adaptability, efficient use of water and land, and ecological cycling [1]. With this background in mind, aquaculture research teams in Henan Province have developed a “168” largemouth bass culture model (Supplementary Figure S1 shows the model diagram). In this model, a highly efficient funnel-shaped recirculating pond aquaculture system is used. With respect to the number “168” in this model, “1” represents a culture area of 1000 m^2^, “6” represents six key technologies, including funnel-based waste collection and ecological recirculation, and “8” indicates eight core advantages, such as water savings and land savings [6]. The core aquaculture unit consists of a circular funnel-shaped tank, complemented by a prefabricated temperature-controlled greenhouse to buffer external temperature fluctuations and maintain a relatively stable microenvironment for aquaculture. The system uses tangentially arranged hydrodynamic equipment to drive the water into a directed vortex flow field, enabling efficient solid–liquid separation of uneaten feed and feces through gravitational settling and pressure differentials caused by overflow. The separated effluent is fully recycled after vertical flow sedimentation, microfiltration, and ecological purification in constructed wetlands, thereby fundamentally cutting off the pathways for non-point source pollution to spread. This structure not only significantly improves the efficiency of water and land resource utilization (water savings exceeding 80% and land savings of about 50%) but also enables resource recovery from aquaculture waste through a “water-waste separation-nutrient recycling” mechanism. Furthermore, leveraging topographical elevation differences can support expanded agro-aquaculture integration models such as “168 + rice/lotus/fruit,” demonstrating strong ecological adaptability and potential for wide application [7]. However, this aquaculture model still faces several challenges. Feedback from the “168” model culture base suggests that as fish grow, the biomass per unit volume increases continuously; fish initially show symptoms of suboptimal physiological status, such as lower feed intake, followed by sporadic mortality. Necropsy of dead individuals revealed enlarged and darkened spleens, local swelling, and whitening of gill filaments, with no specific pathological signs. The research team preliminarily determined that the core cause lies in the fixed area of the farming unit. After the initial fry were introduced, pond segregation and density regulation were not promptly implemented according to the growth stage, resulting in a progressive accumulation of actual stocking density as the fish grew. This progressive high-density stress, caused by “fixed-pond, non-graded” management, can easily exceed the physiological adaptation threshold of the fish, inducing immune system decline and reduced stress resistance, ultimately leading to stunted growth and lower survival rates.

Aquaculturists often implement high-density culture as a key strategy for increasing aquaculture production, especially when water is scarce. However, high stocking density often reduces the carrying capacity of the environment and increases physiological stress, which can hinder fish growth, immunity, metabolism, and intestinal health [8,9]. High-density culture inhibits growth and disrupts physiological homeostasis, leading to elevated stress hormone levels, disordered antioxidant enzyme systems, and downregulated expression of genes associated with the immune system [2,10,11]. High stocking density also causes intestinal damage and microbial dysbiosis, thereby impairing nutrient absorption [12,13]. Stocking density also strongly influences the composition of the gut microbiota; specifically, the abundance of beneficial bacteria decreases, whereas that of opportunistic pathogens increases under high-density conditions [8]. Despite these important findings in various species, systematic research on largemouth bass under a specific “168” recirculating system remains limited. In particular, no study has integrated histology, biochemistry, transcriptomics, and metabolomics to investigate the phasic responses (from compensation to exhaustion) of the liver—the central organ for metabolism, detoxification, immunity, and growth—under increasing stocking densities in this system. This knowledge gap hinders the optimization of stocking density and the development of science-based management practices for the “168” model.

Multiomics analysis integrates transcriptomic and metabolomic data to link genes with metabolites. Many researchers have applied it in aquaculture to elucidate organismal responses to adverse environments. In this study, histological observations, biochemical assays, and integrated omics analyses were performed to investigate how different stocking densities affect the health, metabolism, immune status, and overall physiological condition of largemouth bass cultured under the “168” recirculating aquaculture system. Specifically, this study aimed to elucidate the physiological and molecular mechanisms underlying density-induced changes in growth, metabolism, and immunity. The health of the fish under the “168” aquaculture model was comprehensively assessed, the optimal stocking density range was identified, and practical suggestions for improving this aquaculture system were provided.

## 2. Materials and Methods

### 2.1. Aquaculture Management of the “168” Aquaculture Base

The largemouth bass used in this study was sourced from the “168” Aquaculture Base in Zhongmu County, Zhengzhou City, Henan Province, China; the study area was equipped with multiple recirculating aquaculture systems (RAS). All the largemouth bass fingerlings at this farm were obtained from the same breeding facility to ensure genetic consistency. The fingerlings were stocked in multiple batches; within each pond, all the fingerlings were from the same batch and were uniform in size. Based on the actual stocking density of each pond (assessed by in situ measurement of fish body weight), the ponds were classified into three density groups: LD, MD, and HD. The rearing cycles for the largemouth bass in the three density groups were about 60, 90, and 100 days. The LD group had a stocking density of 2.5 ± 0.5 kg/m^3^, the MD group had a stocking density of 4.0 ± 0.5 kg/m^3^, and the HD group had a stocking density of 7.5 ± 0.5 kg/m^3^. Each pond was fed twice daily, and the feeding rates for different sizes are shown in Supplementary Table S1. To distinguish between the direct effects of stocking density and the potential confounding effects of water quality, daily instantaneous water samples were collected from all the ponds over the 14 days preceding fish sampling. Key water quality parameters (dissolved oxygen, temperature, pH, nitrite and ammonia nitrogen) were measured. All measured water quality parameters were within the suitable range for largemouth bass aquaculture, and the Kruskal–Wallis H test showed that water quality conditions were generally comparable among the three density groups, with only occasional significant differences observed at individual time points (*p* > 0.05) (Supplementary Table S2). During the experiment, dissolved oxygen, temperature, pH, nitrite, and ammonia nitrogen were monitored daily at fixed times as part of the farm’s routine water quality management. All the measured values remained within the suitable ranges for largemouth bass culture and were consistent with the 14-day pre-experimental baseline, confirming that the water quality conditions were stable throughout the study.

### 2.2. Fish Sample Collection

Largemouth bass were sampled from the production ponds at this base. On the sampling day, one pond was randomly selected from each of the LD, MD, and HD groups. Largemouth bass specimens were randomly sampled from each group, with eight individuals collected per group (*n* = 8). The morphological characteristics of the 24 fish are presented in Supplementary Table S3. The average stocking densities were recalculated based on the sampled individuals (Supplementary Table S4) and were consistent with the predefined experimental criteria.

After the fish (*n* = 24) were anesthetized with MS-222 (100–150 mg/L; buffered to neutral pH with sodium bicarbonate), their liver tissues were rapidly excised. The collected tissues were weighed and subdivided into four portions. One portion was fixed in a 4% universal tissue fixative (Cat. No.: P1110; Wuhan Seville Biotechnology Co., Ltd., Wuhan, China) for subsequent histological analysis. The remaining three portions were placed in DNase-free and RNase-free cryogenic vials, immediately snap-frozen in liquid nitrogen, and stored at −80 °C until further analysis. All experimental procedures were approved by the Animal Experiment Ethics Committee of Henan Normal University (Approval No.: HNSD-2026BS-0403). MS-222 was used throughout the experiment to reduce stress and ensure animal safety.

### 2.3. Histopathological Assay

Liver tissues were fixed with a 4% universal tissue fixative and cut into thin histological sections by Wuhan Servicebio Technology Co., Ltd. (Wuhan, China). The procedures included dehydration, clearing, paraffin embedding, sectioning, and H&E staining. The procedures are detailed in Supplementary Note S1. The stained sections were observed and imaged under an upright light microscope (Olympus BX51, Olympus Corporation, Tokyo, Japan) equipped with a digital camera (Zeiss AxioCam MRc, Carl Zeiss, Oberkochen, Germany).

### 2.4. Biochemical Parameters

To evaluate the physiological responses of fish under high-density farming conditions, we assessed a series of biochemical and hormonal indicators, including antioxidant markers (GSH-Px, MDA, and CAT) [14], a growth-related hormone (GH) [15], an immune indicator (IL-1β) [16], and a stress-related hormone (cortisol) [16], as described in previous studies.

Liver tissues stored at –80 °C were thawed, and 0.5 g of tissue was weighed. Physiological saline was added to the samples at a weight-to-volume ratio of 1:9, after which the samples were homogenized under ice-water conditions to obtain a 10% (*w*/*v*) tissue homogenate. This homogenate was divided into two portions for further analysis. One portion was used for the biochemical assays. The protein concentration, activities of CAT and GSH-Px, and content of MDA were determined using commercial assay kits (Nanjing Jiancheng Institute of Biological Engineering, Nanjing, China) following the manufacturer’s instructions. The other portion was used for the enzyme-linked immunosorbent assay (ELISA). The concentrations of cortisol, GH, and IL-1β were quantified using commercial ELISA kits (Shanghai Yuanju Biotechnology Center, Shanghai, China) following the manufacturer’s protocols. Information on the kits used in this study is provided in Supplementary Table S5. All the data were processed and analyzed using GraphPad Prism 10.

### 2.5. Transcriptome and Metabolome Analyses

Liver tissues from the three density groups (LD, MD, and HD) were collected, flash-frozen in liquid nitrogen, and transported on dry ice for analysis. A total of 18 individual fish were used in this study, with six independent biological replicates per density group (*n* = 6 per group). No parallel rearing units or sample pooling was performed, and each fish was processed and sequenced as an independent sample. Transcriptomic sequencing was conducted by Beijing Novogene Technology Co., Ltd., Beijing, China, and untargeted metabolomic analysis was performed via LC–MS by Shanghai Baiqu Medical Technology Co., Ltd., Shanghai, China.

#### 2.5.1. Transcriptomic Analysis

The main steps of the transcriptomic analysis consisted of RNA extraction, library construction, and sequencing. The procedures are detailed in Supplementary Note S2. To ensure that the expression levels of the transcripts were accurately estimated, the number of mapped reads and transcript length were normalized across samples. Gene expression levels were quantified using the software StringTie [17], based on the maximum flow algorithm; FPKM served as the normalization metric. The distribution of gene expression levels among samples was visualized using box plots. PCA was performed to reduce the dimensionality of the samples. Differential expression analysis between groups (LD vs. MD and LD vs. HD) was conducted using the software DESeq2 [18]. Genes with a fold change ≥ 2 and an *FDR* < 0.05 were identified as DEGs. The results were visualized using Venn diagrams and volcano plots. Functional enrichment analysis of the DEGs was performed based on the KEGG database, and the enrichment results were visualized as bubble plots using ClusterProfiler (v4.4.4) [19,20]. All transcriptomic bioinformatics analyses were conducted using the BioMack Cloud Analysis Platform (https://www.biocloud.net/aboutus, accessed on 8 April 2026).

#### 2.5.2. Metabolomic Analysis

After the proteins were precipitated from the samples using an extraction solution, the resulting supernatants were analyzed by UHPLC–MS in both positive and negative ion modes. The raw data were converted and processed using an in-house R-based program for metabolite identification. The procedures are detailed in Supplementary Note S3. Multivariate statistical analyses, including PCA and OPLS-DA, were performed. DEMs were screened based on FC, *p* value, and VIP, with thresholds of *FC* > 1.2, *p* < 0.05, and *VIP* > 1. The data were compared between LD and MD and between LD and HD. KEGG pathway enrichment analysis was performed by conducting a hypergeometric test using the ClusterProfiler (v4.4.4) [19,20], and the results were visualized using bubble plots. All metabolomics analyses were conducted using BioMack Cloud Analysis (https://www.biocloud.net/aboutus, accessed on 8 April 2026).

### 2.6. Association Analysis in Transcriptomics and Metabolomics

Based on the results of the DEG and DEM analyses, the DEGs and DEMs from the same comparison groups were jointly mapped onto KEGG pathways. The relationships among genes, metabolites, and pathways were established by parsing KGML files to construct an integrated network of DEGs, DEMs, and their associated pathways. To determine whether regulatory relationships existed between genes and metabolites, a PCC analysis was performed between all detected genes and metabolites within each comparison group. Before analysis, the data were normalized using Z score transformation. Gene–metabolite pairs with strong correlations were screened based on the thresholds of |*r*| > 0.80 and *p* < 0.05. Next, the significantly correlated DEGs and DEMs were extracted for each pathway, and gene—metabolite correlation networks were constructed. Key nodes were identified based on the resulting networks, and potential regulatory modules were further evaluated in combination with the results of the KEGG pathway enrichment analysis.

### 2.7. Quantitative Real-Time PCR Analysis

Based on the results of the integrated transcriptomic and metabolomic analyses, specific primers were designed using Primer Premier 5 software. Total RNA was extracted from the liver tissues of the fish in the LD, MD, and HD groups (three biological replicates per group) using TRIzol reagent following the manufacturer’s instructions. The extracted RNA was reverse-transcribed into cDNA using a High-Performance qRT Master Mix Kit (Kunming Yungeng Biotechnology Co., Ltd., Kunming, China), with *gapdh* serving as the internal reference gene. The primer sequences are listed in Supplementary Table S6. Next, qRT–PCR was performed using a 2× Universal SYBR qPCR Master Mix Kit (Kunming Yungen Biotechnology Co., Ltd., Kunming, China). Relative gene expression levels were calculated using the 2^−ΔΔCt^ method [21].

## 3. Results

### 3.1. Histological Observation of the Liver

**Figure 1:**
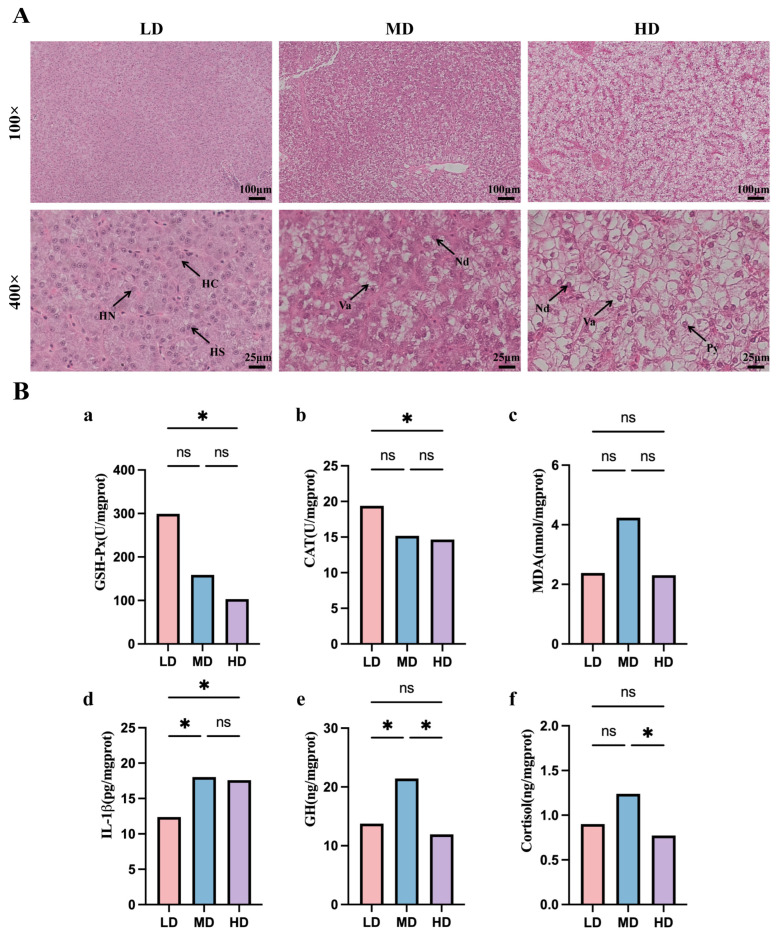
Histological images and biochemical analysis results of the liver of largemouth bass are shown. (**A**): H&E-stained sections. HC: hepatocytes; HS: hepatic sinusoids; HN: cell nucleus; Va: vacuolization; Nd: nuclear displacement; Py: nuclear condensation. Magnification: 100× and 400×. (**B**): Physiological and biochemical parameters. (**a**): GSH-Px; (**b**): CAT; (**c**): MDA; (**d**): IL-1β; (**e**): GH; (**f**): cortisol. *: Indicates a significant difference between groups (*p* < 0.05), ns: not significant (*p* > 0.05).

In the LD group, the liver tissue displayed a regular architecture, with large and round nuclei centrally located, a homogeneous cytoplasm, and prominent hepatic sinusoids between adjacent hepatic cords. As the stocking density increased, hepatocyte vacuolization became more frequent in the MD group and was more severe in the HD group. In the MD group, several vacuoles, nuclear displacement, and disorganized hepatic cords were observed, and lobular structures showed focal fragmentation. In the HD group, vacuolization was further aggravated, the nuclei became condensed with chromatin aggregation, and pyknosis was apparent. Most of the normal lobular architecture was destroyed, and only residual cord-like structures remained (Figure 1A).

### 3.2. Biochemical Parameters

The activities or concentrations of GSH-Px, CAT, MDA, GH, cortisol, and IL-1β in liver tissue varied among different stocking densities (Figure 1B). The levels of GSH-Px and CAT were significantly lower at higher densities (Figure 1(Ba,Bb)). The MDA level was highest in the MD group, although the difference in the MDA content between the MD group and the other densities (LD and HD) was not significant (Figure 1(Bc)). The IL-1β level was highest in the MD group; compared with that in the LD group, it was significantly greater in the MD and HD groups (*p* < 0.05), but the difference between the MD and HD groups was not significant (Figure 1(Bd)). GH and cortisol levels were highest in the MD group. GH levels were significantly higher in the MD group than in the LD group, suggesting compensatory activation, but were significantly lower in the HD group than in the MD group, indicating exhaustion of the growth axis (Figure 1(Be)); cortisol levels followed a pattern similar to that of GH levels, suggesting a comparable exhaustion phase of the stress axis, although the difference in cortisol levels between the LD and MD groups was not significant (Figure 1(Bf)).

### 3.3. Transcriptomic Analysis

**Figure 2:**
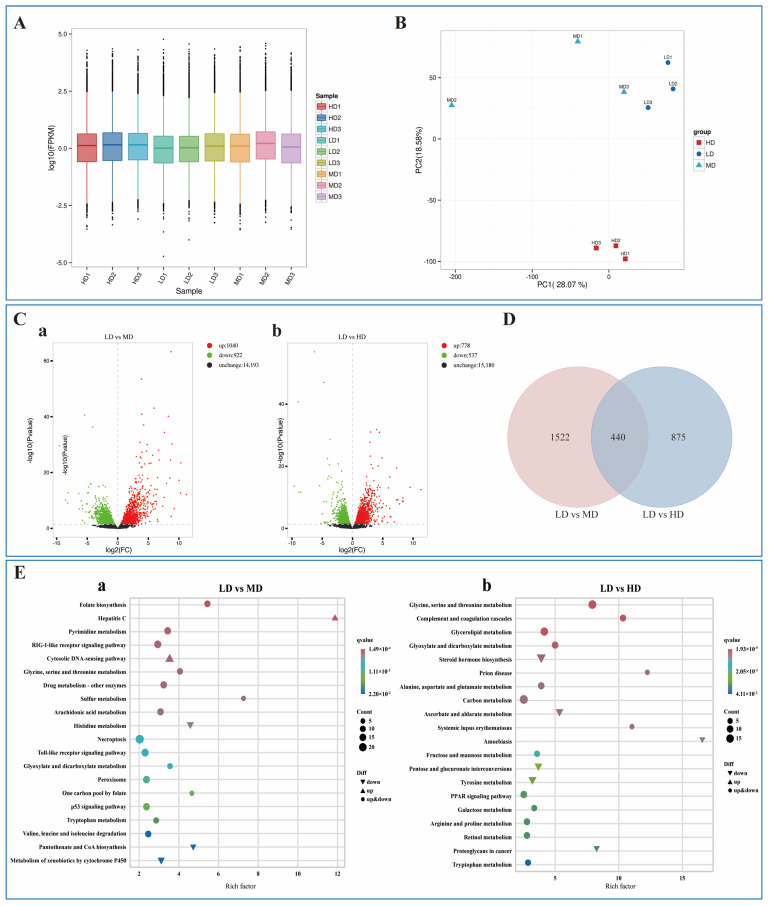
Transcriptomic analysis of largemouth bass under high-density rearing stress. (**A**) Box plots showing the FPKM distributions for each sample. (**B**) Principal component analysis (PCA) of the transcriptome is illustrated. (**C**) Volcano plots showing differentially expressed genes: (**a**) LD vs. MD; (**b**) LD vs. HD. (**D**) Venn diagrams showing DEGs between the LD vs. MD and LD vs. HD groups. (**E**) The KEGG enrichment bubble plots illustrate the top 20 DEGs: (**a**) LD vs. MD; (**b**) LD vs. HD. The y-axis represents the KEGG pathway names, and the x-axis represents the enrichment factor.

Nine samples were used for eukaryotic paired-end transcriptome sequencing, yielding 62.37 Gb of clean data. At least 6.25 Gb of clean reads were generated from each sample, and the percentage of Q30 bases was 96.49% or greater. The mapping efficiency ranged from 95.63–96.71% across samples. The gene expression levels of each sample are detailed in Supplementary Table S7. Box plots of the FPKM values and PCA results revealed tight clustering within each treatment and clear separation among groups, indicating satisfactory biological reproducibility and high data reliability for downstream analysis (Figure 2A,B).

Volcano plots were generated to visualize the overall distribution and statistical significance of changes in gene expression between groups (Figure 2(Ca,Cb)). Many DEGs were detected in both the LD vs. MD and LD vs. HD comparisons. Their distribution was largely symmetric, indicating that high-density stress regulates gene expression bidirectionally; detailed statistical information is provided in Supplementary Table S8. Venn analysis revealed that 440 DEGs were shared by the two comparisons, whereas 1522 DEGs were specific to LD vs. MD, and 875 DEGs were specific to LD vs. HD (Figure 2D). These shared genes may represent core responses to density stress, whereas the group-specific genes may reflect differences in stress intensity.

The results of the KEGG enrichment analysis of the top 20 pathways revealed that the DEGs in the LD vs. MD comparison were enriched mainly in folate biosynthesis, pyrimidine metabolism, glycine, serine, and threonine metabolism, cytosolic DNA sensing, RIG-I-like receptor signaling, sulfur metabolism, arachidonic acid metabolism, and histidine metabolism (Figure 2(Ea)). In the LD vs. HD comparison, the major pathways included glycine, serine, and threonine metabolism; complement and coagulation cascades; glycerophospholipid metabolism; glyoxylate and dicarboxylate metabolism; steroid hormone biosynthesis; carbon metabolism; alanine, aspartate, and glutamate metabolism; prion disease; and ascorbate and aldarate metabolism (Figure 2(Eb)). Combined KEGG enrichment analysis revealed that the DEGs were enriched mostly in amino acid metabolism, immune and stress signaling, and energy metabolism. These findings suggest that these pathways play key roles in the molecular regulatory network under high-density culture stress.

### 3.4. Metabolomics Analysis

**Figure 3:**
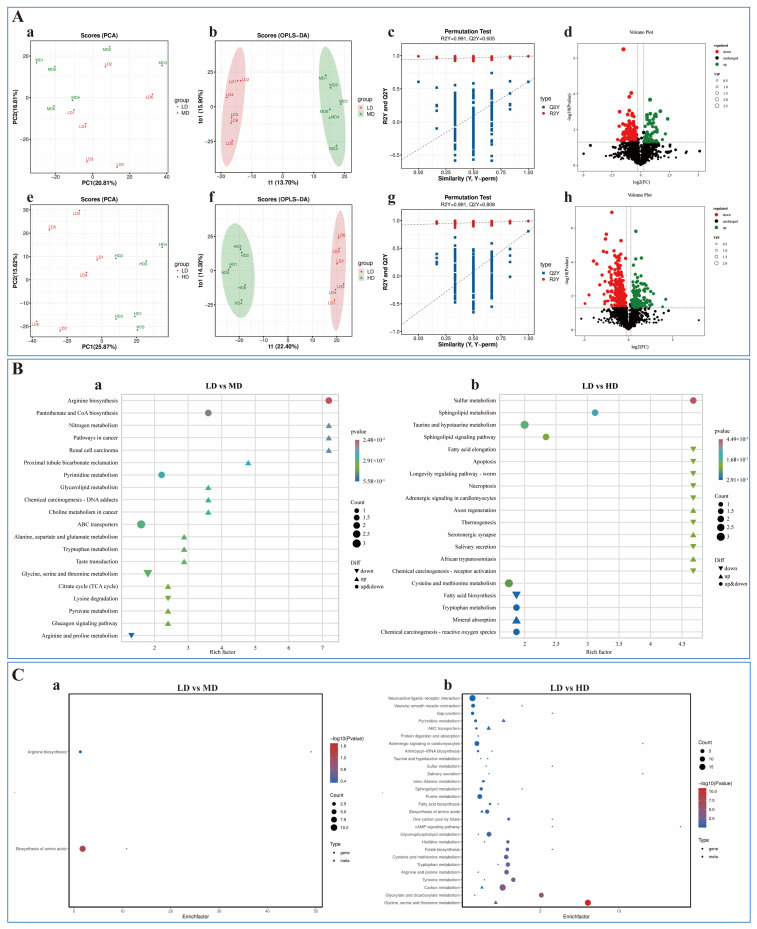
Metabolomic and integrated analyses of largemouth bass under high-density rearing stress are illustrated. (**A**,**B**) Metabolomic analysis of largemouth bass was performed. (**A**): (**a**,**e**): PCA. (**b**,**c**,**f**,**g**): Orthogonal partial least squares discriminant analysis (OPLS-DA). (**b**): OPLS-DA score plot for the LD vs. MD group. (**f**): OPLS-DA score plot for the LD vs. HD group. (**c**): OPLS-DA model replacement test plot for the LD vs. MD group. (**g**): OPLS-DA model replacement test plot for the LD vs. HD group. (**d**): Volcano plot showing the DEMs for the LD vs. MD group. (**h**): Volcano plot showing the DEMs for the LD vs. HD group. (**B**): KEGG enrichment bubble plots for the top 20 DEMs are illustrated. (**a**): LD vs. MD, (**b**): LD vs. HD. The y-axis and x-axis represent KEGG pathway names and enrichment factors, respectively. (**C**): KEGG enrichment bubble plots for DEGs and DEMs are illustrated. (**a**): LD vs. MD, (**b**): LD vs. HD. The y-axis and x-axis represent KEGG pathway names and enrichment factors, respectively.

PCA was first performed as a quality control step for the metabolomic data from the LD, MD, and HD groups. No outliers were detected, but the three groups were not clearly separated in the unsupervised model, indicating that supervised analysis was necessary (Figure 3(Aa,Ae)). OPLS-DA resulted in clear separation between the LD group and the MD and HD groups, suggesting substantial density-dependent changes in the metabolic profile (Figure 3(Ab,Af)). In both comparisons, the R^2^Y values were greater than the Q^2^Y values, and permutation testing revealed no evidence of overfitting, confirming that the model was robust (Figure 3(Ac,Ag)). Using |*FC*| > 1.2, *p* < 0.05, and *VIP* > 1 as the screening criteria, DEMs were identified between groups. In the LD vs. MD comparison, 209 DEMs were detected, including 81 upregulated metabolites and 128 downregulated metabolites. In the LD vs. HD comparison, 496 DEMs were detected, including 146 upregulated metabolites and 350 downregulated metabolites. Thus, as the stocking density increased, the metabolic response shifted from a predominantly activated pattern in the MD group to a predominantly suppressed pattern in the HD group (Figure 3(Ad,Ah)). The results of the KEGG enrichment analysis revealed that the DEMs in the LD vs. MD comparison were associated mainly with arginine biosynthesis, amino acid metabolism, and energy metabolism, whereas the DEMs in the LD vs. HD comparison were enriched mainly in sulfur metabolism, sphingolipid metabolism, sulfur-containing amino acid metabolism, lipid metabolism, and signal transduction pathways (Figure 3(Ba,Bb)). These changes indicate that high density disrupts both amino acid and lipid metabolic homeostasis.

### 3.5. Integrated Analysis of Transcriptomics and Metabolomics

The results of the KEGG enrichment analysis of the shared DEGs and DEMs indicated that the overlap in LD vs. MD was concentrated mainly in amino acid biosynthesis and arginine biosynthesis (Figure 3C). Among these pathways, *glsb* was significantly upregulated, whereas the citrulline level decreased significantly; *nags*, *aco2*, and *aldh18a1* were significantly upregulated, whereas *pfkla* and *psph* were significantly downregulated.

**Figure 4:**
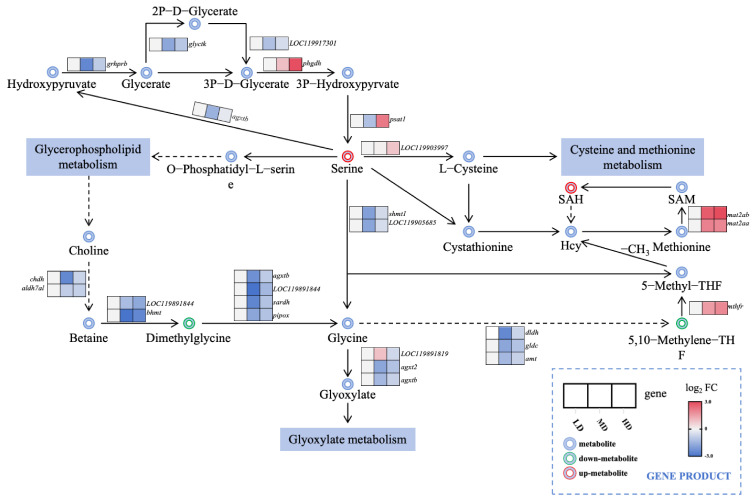
Correlation analysis and regulatory network of DEGs and DEMs in the top five pathways are presented.

In the LD vs. HD comparison, the top enriched pathways included carbon metabolism; glycine, serine, and threonine metabolism; glyoxylate and dicarboxylate metabolism; beta-alanine metabolism; histidine metabolism; amino acid biosynthesis; tryptophan metabolism; folate-mediated one-carbon metabolism; arginine and proline metabolism; and tyrosine metabolism. Among these, carbon metabolism, glycine, serine, and threonine metabolism, and glyoxylate and dicarboxylate metabolism were associated with the greatest numbers of matched genes and metabolites, with Ser, 5,10-mTHF, DMG, and SAH showing substantial overlap, forming a connected regulatory network in which Ser metabolism plays a central role (Figure 4). Ser biosynthesis was enhanced through the upregulation of *phgdh* and *psat1*, whereas its conversion to Gly and the choline-related branch were inhibited via the downregulation of *shmt1*, *agxtb*, *chdh*, *aldh7a1*, and *bhmt*, leading to the accumulation of Ser, which was redirected toward the cystathionine pathway through the upregulation of *LOC119903997* to support antioxidant defense. Concurrently, the reduced availability of methyl donors promoted the accumulation of SAH, and the methionine (Met) remethylation cycle became dysfunctional: the decrease in 5,10-mTHF weakened the remethylation of Hcy to Met, and despite the upregulation of *mthfr*, *mat2ab*, and *mat2aa*, the depletion of methyl donors rendered these efforts ineffective, resulting in the accumulation of SAH. In the arginine and proline pathways, the expression of *gamt*, *oat*, *odc1*, and *smox* was significantly altered, indicating that Arg was preferentially directed toward the NO synthesis pathway rather than the creatine synthesis pathway. The conversion of Arg to ornithine was inhibited by the downregulation of *LOC119884194* and *LOC119915880*, leading to abnormal partitioning of ornithine metabolism: the downregulation of *gamt* reduced creatine synthesis to conserve methyl donors, the downregulation of *oat* weakened proline-dependent antioxidant capacity, and the upregulation of *odc1* promoted putrescine synthesis. Polyamine metabolism was further disrupted, as *smox* and *aco2* were upregulated, catalyzing the conversion of polyamine to β-aminopropionaldehyde, whereas key genes responsible for its conversion to β-alanine (*aldh16a1* and *aldh7a1*) were downregulated, leading to the accumulation of β-aminopropionaldehyde. Additionally, the uridine degradation pathway was suppressed because of the downregulation of *dpydb*, *dyps*, and *upb1*, which reduced 5,6-dihydrouracil and β-alanine production, whereas the downregulation of *abat* and *aldh6a1* further blocked the production of acetyl-CoA and fatty acid biosynthesis. Additional changes in histidine and tyrosine metabolism further supported the coordinated reprogramming of energy metabolism and immune regulation. The core regulatory network is shown in Supplementary Figure S2.

Information on the partial functional annotation of the 14 differentially expressed LOC genes identified in this study is provided in Supplementary Table S9.

### 3.6. Validation of Gene Expression

**Figure 5:**
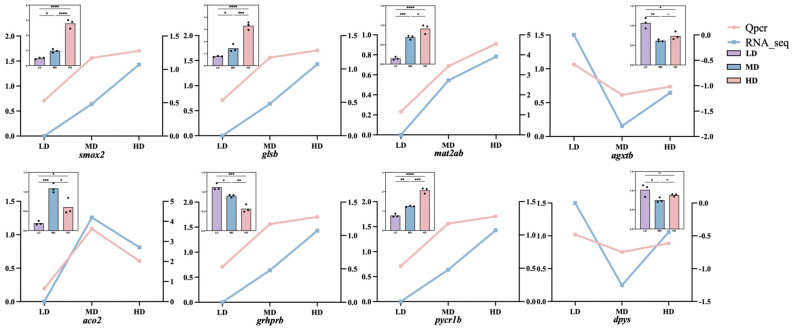
Quantitative real-time polymerase chain reaction (qRT–PCR) was performed for transcriptome validation, ns: not significant (*p* > 0.05); *: *p* < 0.05; **: *p* < 0.01; ***: *p* < 0.001; ****: *p* < 0.0001.

To verify the transcriptomic results, eight genes were selected for qRT–PCR analysis. The expression patterns obtained by qRT–PCR were consistent with the RNA-seq FPKM patterns, and the differences were significant, confirming that the sequencing analysis was reliable (Figure 5).

## 4. Discussion

Largemouth bass is an important freshwater aquaculture species in China. As water scarcity becomes more prevalent, researchers need to promote and optimize high-efficiency recirculating aquaculture systems, such as the “168” aquaculture model, for the production of largemouth bass. However, sporadic mortality associated with high-density farming is a major problem in this system. In high-density environments, limited space and food intensify competition and aggressive behavior, directly increasing mortality [22]. Additionally, high stocking density combined with improper feeding practices leads to the accumulation of metabolic waste and undigested feed, resulting in the deterioration of water quality, which compromises fish health and ultimately decreases survival rates [23]. The liver plays key roles in metabolism, detoxification, immunity, digestion, and growth [11,24,25,26]. Its function directly affects the survival rate, growth performance, and disease resistance of fish [27], making it an ideal target for investigating the effects of increasing stocking density on fish health. In this study, integrated transcriptomic and metabolomic analyses were performed to investigate changes in key genes and metabolites in the liver of largemouth bass under different stocking densities. By combining histological observations with physiological and biochemical measurements, a comprehensive chain of evidence was established to evaluate the health status of largemouth bass reared under different densities within the “168” aquaculture model.

### 4.1. Severe Histopathological Damage in the Liver of Largemouth Bass Under High-Density Culture Stress

Liver vacuolization is a hallmark pathological indicator of environmental stress in fish. Under conditions such as hypoxia, bacterial infection, or high stocking density, hepatocytes may exhibit vacuolization, nuclear displacement, and cell rupture because of metabolic disturbance, energy depletion, or oxidative injury [28,29]. In a study on high-density stress in Jifu tilapia [28], typical vacuolization and nuclear marginalization were observed, and a significant increase in hepatocyte apoptosis was confirmed by TUNEL staining. From a histological perspective, vacuolization in fish hepatocytes may originate from two distinct pathological mechanisms: lipid accumulation (steatosis) or glycogen deposition (glycogenic hepatopathy). Studies have shown that in carnivorous fish such as the largemouth bass, high-carbohydrate diets primarily induce glycogen deposition rather than lipid accumulation within hepatocytes [30]. Notably, H&E staining cannot effectively distinguish between these two types of vacuolization, as both lipids and glycogen are dissolved during tissue processing and appear as transparent vacuoles under microscopic observation. In this study under the “168” rearing model, increased stocking density led to the progressive aggravation of hepatic vacuolization, gradual blurring of cell boundaries, nuclear displacement and atrophy, and progressive disruption of hepatic cord architecture in largemouth bass, indicating that high stocking density causes severe damage to liver tissue. However, the specific cause of hepatic vacuolization in this study remains to be further investigated.

### 4.2. Phasic Responses of Hepatic Biochemical Indices in Largemouth Bass Under High-Density Culture Stress

The activities or concentrations of GSH-Px, CAT, and MDA are key indicators of antioxidant capacity. GSH-Px catalyzes ROS removal via GSH, protecting the integrity of the cell membrane [31], while CAT decomposes hydrogen peroxide into water and oxygen, acting synergistically with GSH-Px [32,33]. Long-term high-density aquaculture comprehensively decreases antioxidant enzyme activities, and the results of this study are consistent with findings in turbot [34], both of which revealed that high-density stress significantly decreases hepatic antioxidant enzyme activities; specifically, the activities of SOD, CAT, and GSH-Px, as well as GSH levels, decrease as density increases. MDA reflects oxidative damage; when ROS attack membrane polyunsaturated fatty acids, lipid peroxidation is triggered, leading to the production of MDA [35], which directly indicates membrane damage [36]. In this study, the MDA content initially increased but then decreased with increasing density, potentially because increased ROS levels and decreased antioxidant enzyme activity during early rearing (low-to-medium density) led to an increase in the MDA content. During later rearing (medium-to-high density), severe liver damage may have reduced the release of MDA, while compensatory ROS clearance contributed to its decrease. Overall, the levels of MDA did not significantly differ, and similar findings in Siberian hybrid sturgeon (*Acipenser baerii ♀* × *Acipenser schrenckii* ♂) revealed unchanged levels of MDA, although the activities of SOD and GSH-Px changed significantly [37]; these findings indicate that multiple indicators are needed to assess oxidative status. Thus, the absence of significant differences in some antioxidant indicators does not imply that oxidative stress is absent but may reflect the action of compensatory mechanisms, species-specific responses, or threshold effects.

IL-1β, a key member of the interleukin-1 (IL-1) family and an important pro-inflammatory cytokine, was selected as a physiological indicator of immune status. IL-1β serves as an early signaling molecule in the fish immune response [37]. Its expression is generally upregulated during the early stages of infection to activate immune cells, trigger the MAPK pathway, and induce downstream factors [38,39,40]. IL-1β levels are tightly regulated by negative feedback mechanisms to prevent excessive inflammation [41]. Our study revealed that high stocking density significantly upregulated IL-1β levels in largemouth bass, indicating that crowding stress induces an inflammatory response. Consistent with our findings, studies on grass carp (*Ctenopharyngodon idella*) and Nile tilapia (*Oreochromis niloticus*) have shown that high-density stress induces the upregulation of the expression of the pro-inflammatory cytokine IL-1β [28,42]. In contrast, studies on large yellow croaker (*Larimichthys crocea*) revealed that short-term high-density stress led to sustained upregulation of immune-related genes, whereas long-term high-density stress downregulated immune-related genes such as IL-1β, thereby impairing immune defense [43]. These findings suggest that prolonged high-density stress may induce immunosuppression in organisms. In our study, the IL-1β levels were slightly lower in the HD group than in the MD group. On the basis of the findings of this study on large yellow croaker, we hypothesized that this trend may reflect a gradual transition of the immune system from an activated state to a suppressed state during high-density rearing. These findings collectively suggest that the effect of high-density stress on immune function in largemouth bass is characterized by short-term activation followed by long-term suppression.

GH is a peptide hormone secreted by the anterior pituitary gland that promotes the growth and division of somatic cells and is essential for growth, metabolism, and homeostasis in fish [44]. GH promotes the proliferation and differentiation of cells either through direct interaction with the GH receptor or via the GH-IGF-I axis [45]. In African catfish (*Clarias gariepinus*), the expression level of the GH gene decreased significantly after 60 days of rearing at a high density of 125 kg/m^3^, indicating that the growth axis is impaired under prolonged high-density stress [46]. In this study, the GH concentration first increased but then decreased as the stocking density increased. A study on juvenile thick-lipped gray mullet (*Chelon labrosus*) revealed that when individuals were maintained under high-density (6.7 kg/m^3^) rearing conditions, the expression of GH and downstream IGF-I was significantly upregulated. The authors suggested that although the HPI axis suppresses growth by inhibiting the GH-IGF-I axis under high-density conditions, the organism simultaneously activates the growth axis to compensate for growth suppression, indicating that both axes play a role in regulating metabolic and stress responses under different rearing densities [45]. In our study, GH levels were significantly higher in the MD group than in the LD group, whereas GH levels were significantly lower in the HD group than in the MD group. This pattern may be due to a compensatory response in the MD group, in which the growth axis was activated for a short duration under moderate density stress. In contrast, under prolonged high-density stress, a shift from compensation to impairment of the growth axis may have occurred; similar observations were made in African catfish [46].

Cortisol is the primary glucocorticoid in fish and exhibits both glucocorticoid and mineralocorticoid activities. It plays a central role in stress responses, metabolic regulation, and immune modulation [47,48]. Serum cortisol levels are a reliable indicator of stress levels in fish. The liver is the primary organ responsible for cortisol metabolism and clearance, and the cortisol content in the liver reflects the rate of cortisol uptake and metabolic activity [49]. In humans, adrenal insufficiency is characterized by insufficient secretion of cortisol; affected individuals cannot produce adequate amounts of hormones even under stress. Similarly, in studies on yellowtail amberjack (*Seriola lalandi*), although serum cortisol levels consistently increased with increasing stocking density over 60 days, GH and IGF-I levels were suppressed, suggesting that a transition may have occurred from a “compensatory” state to an “exhausted” state [50]. In this study, the significant reduction in the cortisol concentration in the HD group may be attributable to the depletion of inter-renal tissue. However, as hepatic cortisol content is regulated by multiple factors, including uptake and metabolism, further investigation is needed to clarify the underlying regulatory mechanisms involved.

### 4.3. Metabolic Reprogramming and Methyl Donor Depletion Induced by High-Density Stress

In this study, the molecular response mechanisms in the liver of largemouth bass under high-density rearing stress were preliminarily investigated by integrating transcriptomic and metabolomic analyses. We found that the metabolic regulatory patterns of the fish were distinct when they were reared at different densities. The DEGs and DEMs between the LD and MD groups were relatively dispersed, and correlation analysis of the amino acid biosynthesis and Arg biosynthesis pathways suggested possible impairment of the urea cycle in the MD group. In response, the urea cycle appeared to be partially reactivated in this group, accompanied by increased Glu acetylation and a possible redirection of metabolic flux toward Arg and Pro synthesis, which may facilitate ammonia clearance and support the repair of damaged hepatocytes. Consistent with the metabolic reprogramming observed in the MD group, studies on Senegalese sole (*Solea senegalensis*) and Asian seabass (*Lates calcarifer*) have confirmed that high-density rearing conditions induce hepatic ureagenesis through the upregulation of glutaminase, arginase, and GDH activities, concurrently promoting arginine consumption and ammonia detoxification [51,52]. By investigating the associations between key DEGs and DEMs involved in key metabolic pathways in the comparison between LD and HD, we aimed to elucidate the mechanisms underlying the response of largemouth bass to high-density stress. Integrated metabolic pathway analysis revealed that the growth rate of fish in the HD group was significantly reduced, which likely served to minimize energy expenditure, and that more energy was redirected toward antioxidant pathways as an adaptive strategy. Similarly, a study on high-density farming of gilthead seabream (*Sparus aurata*) [53] reported that fish in the high-density group (25 kg/m^3^) exhibited significantly lower feed intake and a lower growth rate, accompanied by a reduction in the respiration-to-swimming activity ratio, as determined by loggers’ AE-FishBIT data. These findings indicate that less energy was allocated to growth under high-density conditions.

Correlation analysis of core pathways, including carbon metabolism, glycine metabolism, and folate-mediated one-carbon metabolism, revealed that Ser synthesis increased significantly in the HD group, leading to its accumulation. However, excess Ser did not appear to enter the choline-Gly metabolic branch; instead, it seemed to be preferentially shunted toward the cystathionine pathway to reinforce antioxidant defenses—a shift that may represent an adaptive effort to compensate for methyl donor insufficiency. Nevertheless, this rerouting appeared insufficient to fully preserve antioxidant capacity, which was ultimately compromised, most likely as a consequence of ongoing methyl donor deficiency. This regulatory shift may have occurred because of the redirection of Ser toward the Met remethylation cycle, which became dysfunctional because of methyl donor deficiency, culminating in significant accumulation of SAH. Several studies have validated this metabolic pattern across multiple species; for example, in zebrafish (*Danio rerio*) ahcy mutants, SAH accumulation is associated with mitochondrial dysfunction and oxidative stress [54]; in fish Met metabolism, Ser and Gly promote the conversion of Hcy to Cys for GSH synthesis [55]; and in anurans, the integration of Ser metabolism with one-carbon metabolism coordinates antioxidant defense and methylation reactions [56]. These studies suggest a potential molecular link between SAH accumulation caused by methyl donor deficiency and impaired antioxidant capacity, providing a mechanistic explanation for the cost associated with Ser metabolic redirection under high-density stress.

Correlation analysis of β-alanine metabolism, along with arginine and proline metabolism as two core pathways, revealed that in largemouth bass from the HD group, methyl donors appeared to be conserved through the inhibition of creatine synthesis, whereas arginine seemed to be preferentially directed toward NO synthesis to counteract oxidative stress. Concurrently, the downregulation of uridine-derived β-alanine synthesis, coupled with the dysregulation of polyamine metabolism, appeared to promote the intracellular accumulation of β-aminopropionaldehyde, which may have prompted cells to restrict energy supply. These metabolic patterns are consistent with findings from other species. For example, in rainbow trout (*Oncorhynchus mykiss*), high-density stress prioritizes arginine flux toward NO over creatine [57]; in two-spot catfish (*Ompok bimaculatus*), polyamine pathways undergo significant remodeling under high-density conditions, with putrescine accumulation closely associated with tissue oxidative damage [58], and in zebrafish, downregulation of dpydb and upb1 directly decreases the synthesis of β-alanine, affecting coenzyme A production and fatty acid metabolism (https://www.kegg.jp/entry/dre00410) (8 April 2026). These studies collectively suggest that the reprogramming of arginine and proline metabolism, the dysregulation of polyamine metabolism, and impaired β-alanine synthesis may be key features of metabolic adaptation in fish livers under high-density stress.

Moreover, coordinated downregulation of β-alanine, His, and tyrosine metabolism suggested broader suppression of energy metabolism and immune regulation, with detailed pathway disruptions illustrated in Supplementary Note S4.

### 4.4. Multi-Dimensional Metabolic Adaptation Mechanisms in the Liver of Largemouth Bass Under High-Density Stress

By integrating transcriptomic and metabolomic data, we revealed that the metabolic regulatory network in the liver of largemouth bass exhibited distinct phasic characteristics in response to stocking density-induced stress. These dynamic molecular changes form a potential chain of evidence linking the process underlying pathological damage to the phasic responses of biochemical indicators.

Under MD-induced stress, the core metabolic conflict in the liver appeared to indicate a relative insufficiency in the capacity to process nitrogen waste. Greater glutamine breakdown suggested an increase in the ammonia load, but the significant decrease in the concentration of citrulline, a key intermediate in the urea cycle, directly indicates that the flux of this cycle is impaired. This molecular-level “urea cycle bottleneck” aligns with classical insights into the stress physiology of fish. In a study on ammonia-induced stress in yellow catfish (*Pelteobagrus fulvidraco*) [59], RNAi experiments confirmed that when the urea cycle is impaired, glutamine synthesis is activated to counteract the toxic effects of ammonia, and the expression of key enzymes associated with the urea cycle significantly changes under ammonia stress, suggesting that the urea cycle is a sensitive target for fish in response to ammonia stress. Under acute ammonia stress, the liver of yellow catfish promotes ammonia clearance by upregulating genes related to the urea cycle, while the glutamine synthesis pathway is also activated to synergistically counteract the toxic effects of ammonia [60]. In our study, in response to this bottleneck, the liver appeared to initiate a bidirectional compensatory mechanism. On the one hand, it seemed to allosterically activate the rate-limiting enzyme of the urea cycle by increasing Glu acetylation. Glu acetylation is a key node in the regulation of urea cycle flux, and the allosteric regulatory function of NAGS is highly conserved in vertebrates; hepatic upregulation in response to nitrogen metabolic load represents a conserved adaptive mechanism [61]. On the other hand, metabolic flux appeared to be redirected toward the synthesis of Pro and Arg. Studies on ammonia exposure in large-scale loaches (*Paramisgurnus dabryanus*) [62] have shown that high levels of ammonia trigger liver-specific amino acid metabolic reprogramming in fish, redirecting nitrogen metabolism toward less toxic amino acid derivatives to mitigate the toxic effects of ammonia. This “diversion” strategy aligns with histological observations—since Pro serves as a precursor for collagen synthesis, the upregulation of its synthetic pathway may have suggested a greater demand for liver tissue repair (the MD group exhibited significant vacuolization and disorganized cellular arrangement). However, the overall inhibition of the Ser-Gly metabolic pathway at this stage suggested that resources in these fish were preferentially allocated toward maintaining nitrogen homeostasis rather than supporting methylation reactions. A comparative metabolic study on vertebrate livers [63] revealed that the catabolic processes of Ser and Gly simultaneously produce free ammonia; inhibition of this pathway directly decreases endogenous ammonia production. Biochemical indicators revealed a significant increase in IL-1β levels in the MD group, whereas GH levels exhibited a compensatory increase, indicating that the immune and growth axes were in an activated state and suggesting that localized metabolic disturbances at the molecular level were still within the overall homeostatic regulatory capacity of the liver.

Under HD stress, metabolic regulation in the liver appeared to undergo a fundamental shift, with antioxidant defense becoming the primary focus; however, this adaptation seemed to decrease overall methylation capacity and deplete energy reserves. In this study, histological examinations revealed that hepatocytes had already sustained severe damage, including nuclear pyknosis and disruption of hepatic cord architecture, suggesting that the organism was transitioning from a compensatory state toward exhaustion. The most striking feature was the reorientation of Ser metabolism. Ser synthesis increased significantly, but its downstream pathways were reprogrammed: the pathway leading to one-carbon unit metabolism was inhibited, the choline-Gly metabolic branch was comprehensively downregulated, and the transsulfuration pathway leading to cystathionine-Cys-GSH was activated. In a mouse model of hepatic steatosis [64], *shmt2* activity was found to be directly correlated with the availability of Gly. Excessive activation of *shmt2* led to the depletion of Gly, which in turn impaired GSH synthesis and exacerbated oxidative stress-induced liver injury; in contrast, inhibition of *shmt2* or supplementation with Gly effectively restored GSH levels and alleviated liver injury. The rationale behind this redirection is clear: under high oxidative stress, the liver appeared to sacrifice methylation reactions and phospholipid synthesis, redirecting limited Ser toward GSH synthesis to maintain oxidative balance. This may explain why, although the activity of GSH-Px and CAT was reduced, the MDA level did not increase significantly—the compensatory redistribution of resources within the antioxidant system maintained oxidative homeostasis for a certain period. Similarly, in a study on high-density turbot aquaculture [34], antioxidant enzyme activity decreased without a corresponding increase in oxidative damage markers, and the researchers attributed this to metabolic adaptation rather than a simple reduction in antioxidant capacity.

However, this adaptation appeared to result in severe side effects, including methyl donor deficiency and decreased global methylation capacity. Owing to the reduced flux of serine into one-carbon metabolism, insufficient 5,10-methylenetetrahydrofolate is generated, which may lead to a shortage of methyl supply for the methionine remethylation cycle, resulting in the accumulation of the methyltransferase inhibitor SAH. The methionine cycle serves as a central hub connecting methylation metabolism, antioxidant defense, and energy homeostasis; its dysfunction has far-reaching consequences [65,66]. At high levels, SAH inhibits many methylation-dependent biological processes, including DNA/RNA methylation, phospholipid synthesis, creatine synthesis, and neurotransmitter metabolism. This trade-off strategy has also been observed in other fish species. For example, in Nile tilapia, hepatic phospholipid remodeling—a process that is highly dependent on methyl supply—is a key metabolic adaptation to density stress [67]; in Schlegel’s rockfish (*Sebastes schlegelii*), high-density stress significantly decreases the levels of GH and IGF-I, suggesting that suppression of the growth axis may be related to epigenetic changes caused by decreased methylation capacity [68]. Additionally, further disruptions in polyamine metabolism may increase this cost. In the Arg biosynthetic pathway, upregulation of ODC drives the synthesis of putrescine, potentially representing a compensatory repair attempt in response to severe tissue damage (nuclear pyknosis and disrupted hepatic cords). However, impaired downstream metabolism leads to the accumulation of the cytotoxic substance β-aminopropionaldehyde, whereas inhibition of the uridine degradation pathway reduces the production of β-alanine, ultimately decreasing the acetyl-CoA supply and fatty acid synthesis. This may indicate that, under a strategy prioritizing antioxidant defense, the energy reserve capacity of the liver and the ability of the liver to clear metabolic waste simultaneously decrease. This phenomenon aligns with the findings of Zahedi et al. (2022), who reported that polyamine metabolism disorders are closely associated with tissue damage and that abnormalities in these pathways serve as key indicators of the transition from compensatory function to exhausted physiological function [69].

To summarize, we speculate that the metabolic adaptation of the liver of largemouth bass under high-density stress can be summarized as follows: to prioritize antioxidant defense, these fish seem to redirect key resources such as Ser toward GSH synthesis by sacrificing one-carbon metabolism and methylation reactions, thereby maintaining oxidative balance. However, this strategy appears to be associated with potential systemic methyl donor depletion, disruption of polyamine metabolism, and impaired energy production. Although this “robbing Peter to pay Paul” type of metabolic reprogramming allowed cells to survive in the short term and prevented comprehensive deterioration of biochemical indicators, in the long term, this may deplete the methylation capacity and energy reserves of the liver. Histologically, this state of depletion manifests as severe damage characterized by nuclear pyknosis and disruption of hepatic cord architecture. Similar findings have been reported across species at the mechanistic level; for example, large yellow croaker shifts from an activated state to an inhibited state under high-density stress [43]; and in brook trout (*Salvelinus fontinalis*), alterations in hepatic cortisol metabolism reflect stress-induced exhaustion [70].

### 4.5. Comprehensive Health Assessment and Recommendations for Improving Farming Model

The fish in the MD group appeared to exhibit active compensation. Histological observations revealed early reversible damage, such as hepatocyte vacuolization. Biochemical analyses revealed a significant increase in IL-1β and GH levels, a mild increase in cortisol levels, and no significant changes in antioxidant parameters. Integrated omics analyses suggested that despite impairment of the urea cycle, the body may reactivate it by increasing Glu acetylation and rerouting flux toward Pro and Arg synthesis, suggesting an increased demand for hepatic repair while the antioxidant system appears to maintain oxidative balance through metabolic reprogramming. This speculation is consistent with findings in yellow catfish (*Pelteobagrus fulvidraco*), where activation of autophagy was shown to increase the urea cycle and glutamine synthesis, thereby promoting ammonia detoxification [71]. It also aligns with observations in rainbow trout (*Oncorhynchus mykiss*), in which arginine was redirected toward glutamate and proline synthesis as a countermeasure against heat stress [72].

In contrast, we speculate that the fish in the HD group experienced functional exhaustion. Histological observations revealed irreversible damage, including nuclear pyknosis and disruption of hepatic cords. Biochemically, the decrease in GH levels may reflect impairment of the growth axis. Compared with those in the MD group, the IL-1β levels in the MD group may indicate immunosuppression. Additionally, cortisol levels decreased substantially, suggesting stress axis exhaustion. GSH-Px activity and CAT activity continued to decrease, while the MDA level did not significantly change. On the basis of the results of the transcriptomic and metabolomic analyses, we speculate that, in an attempt to maintain oxidative balance, the body initiates Ser metabolic reprogramming, diverting more Ser to the cystathionine-Cys-GSH pathway. This adaptation appeared to come at the cost of reduced methylation capacity, which may have led to the accumulation of SAH, disruption of polyamine metabolism, and impairment of energy metabolism. This speculation is supported by related studies. A study on rainbow trout (*Oncorhynchus mykiss*) confirmed the association between antioxidant defense and impaired energy metabolism under high-density stress [73], while a study on zebrafish (*Danio rerio*) also confirmed that oxidative stress induces a polyamine stress response and suppresses the urea cycle [74], which is consistent with the speculation of the present study.

These findings suggest that controlling stocking density is necessary to prevent functional exhaustion. Under MD conditions, fish remain in a compensatory state, with active immune and stress responses; this state effectively balances production efficiency with health. Although aquaculture under HD conditions increases yield per unit area, it compromises health, potentially contributing to an increased risk of exhaustion and mortality. IL-1β, GH, and cortisol are sensitive to changes in density and can serve as routine indicators to detect the transition from compensation to exhaustion.

Based on these density-related insights, we propose targeted improvements for the “168” funnel-shaped recirculating aquaculture system for the largemouth bass. First, feeding should be optimized with precise multiple-feeding strategies and feed additives (methyl donors and antioxidants) to reduce residue and alleviate stress. Second, water quality management should be improved by combining physical and biological methods to remove waste. Third, aeration should be improved by installing additional devices in the lower pond to ensure that sufficient oxygen levels are present. Overall, the “168” model balances production efficiency and fish health under MD conditions. Timely pond separation, multi-index monitoring, and nutritional fortification are necessary to achieve healthy and efficient culture.

Notably, this study was conducted at a single “168” aquaculture base; thus, true pond-level replication was not achieved. Nevertheless, the consistency of our findings across multiple analytical approaches strengthens the robustness of the conclusions. Replication across multiple ponds or farms would provide a more solid foundation for extrapolating these findings to the wider “168” production system.

## 5. Conclusions

In this study, the health status of largemouth bass under the “168” aquaculture model was systematically evaluated via histopathological examination, biochemical analysis, and integrated transcriptomic and metabolomic analyses. We found that in the HD group, hepatic metabolism shifted toward reduced methylation capacity and energy reserves to maintain oxidative balance. Biochemical indicators revealed decreased GH levels and reduced GSH-Px and CAT activities, with no significant change in MDA levels, suggesting that metabolic reprogramming may have had a transient protective effect. However, histological findings, including nuclear pyknosis and disrupted hepatic cords, suggested a transition from compensation to exhaustion, which may be associated with the occurrence of sporadic mortality in the HD group. The physiological response was phasic: MD induced compensatory activation of the immune and growth axes, whereas HD led to immune suppression, growth impairment, and metabolic adaptation. The health status of the largemouth bass under the “168” model is density dependent. Rearing under MD conditions appears to promote compensatory adaptation, whereas prolonged rearing under HD conditions causes tissue damage and functional exhaustion. Thus, MD rearing is recommended, along with measures to mitigate HD stress and potentially reduce the risk of mortality.

## Data Availability

The transcriptome data have been deposited in the National Center for Biotechnology Information (NCBI) Sequence Read Archive (SRA) under accession number PRJNA1440993. The data will be made available on request. The metabolomics data have been deposited in the OMIX database at the National Center for Bioinformation (CNCB) under accession number OMIX016056. The data will be made available on request.

## Supplementary Materials

The following supporting information can be downloaded at: https://www.mdpi.com/article/10.3390/ani16132099/s1, Note S1. Paraffin Sectioning and H&E Staining, Note S2. Sample Preparation and Sequencing for Transcriptomic Analysis, Note S3. Sample Preparation and Metabolite Identification for Metabolomics Analysis, Note S4. Integration of β-alanine, histidine, and tyrosine metabolism with immune regulation under high-density stress, Figure S1. Diagram of the 168 Aquaculture Model, Figure S2. Core regulatory network of DEGs and DEMs in histidine metabolism, Table S1. Feeding rates of largemouth bass at the “168” Aquaculture Base, Table S2. Water Quality Indicators 14 Days before Sampling, Table S3. Measurement Results of Morphological Indicators, Table S4. Average stocking density of largemouth bass, Table S5. Summary of kit information, Table S6. Sequences of primers used for qRT-PCR in this study, Table S7. Transcriptome Sequencing Data Statistics, Table S8. Summary of DEGs, Table S9. Functional annotation of LOC genes. References [58,67,69,75,76,77,78] are cited in the Supplementary Materials.

## Abbreviations

**Abbreviation**	**Definition**	**Abbreviation**	**Definition**
LD	Low Density	Ser	Serine
MD	Medium Density	Gly	Glycine
HD	High Density	Glu	Glutamate
H&E	Hematoxylin-Eosin	Arg	Arginine
HC	Hepatocytes	Met	Methionine
HS	Hepatic Sinusoids	Pro	Proline
HN	Cell Nucleus	Cys	Cysteine
Va	Vacuolization	His	Histidine
Nd	Nuclear Displacement	Hcy	Homocysteine
Py	Nuclear Condensation	RNAi	RNA Interference
CAT	Catalase	DMG	Dimethylglycine
GSH-Px	Glutathione Peroxidase	RNA-seq	RNA Sequencing
MDA	Malondialdehyde	NO	Nitric Oxide
GSH	Glutathione	FC	Fold Change
SOD	Superoxide Dismutase	VIP	Variable Importance In Projection
IL-1β	Interleukin-1β	PCC	Pearson Correlation Coefficient
ROS	Reactive Oxygen Species	ANOVA	Analysis Of Variance
GH	Growth Hormone	MAPK	Mitogen-Activated Protein Kinase
qRT-PCR	Quantitative Real-Time PCR	TUNEL	Terminal Deoxynucleotidyl Transferase DUTP Nick End Labeling
SAH	S-Adenosylhomocysteine	AEFishBIT	Acoustic Encoded Fish Biotelemetry Integrated Transmitter
5,10-mTHF	5,10-Methylenetetrahydrofolate	FPKM	Fragments Per Kilobase Of Transcript Per Million Fragments Mapped
KGML	KEGG Markup Language	PCA	Principal Component Analysis
IGF-I	Insulin-Like Growth Factor I	DEGs	Differentially Expressed Genes
HPI	Hypothalamic-Pituitary-Interrenal	DEMs	Differentially Expressed Metabolites
ELISA	Enzyme-Linked Immunosorbent Assay	KEGG	Kyoto Encyclopedia Of Genes And Genomes
LC–MS	Liquid Chromatography-Mass Spectrometry	GH-IGF-I	Growth Hormone-Insulin-Like Growth Factor I
UHPLC-MS	Ultra-High Performance Liquid Chromatography-Mass Spectrometry	OPLS-DA	Orthogonal Projections To Latent Structures-Discriminant Analysis
