# Supplementary material for: Evaluation of the Health Status of Largemouth Bass (Micropterus salmoides) at Different Stocking Densities Under the “168” Aquaculture Model Based on an Integrated Analysis of Liver Histology, Biochemistry, Transcriptomics, and Metabolomics Data
Source: Animals (Basel). 2026 Jul 7;16(13):2099. doi: 10.3390/ani16132099 (PMC13360063; doi:10.3390/ani16132099)
Supplement: Supplementary file 1 [file animals-16-02099-s001.zip › animals-4369566-supplementary.pdf]

Supplementary Information（SI）

Evaluation of the health status of largemouth bass (*Micropterus salmoides*) at different stocking densities under the "168" aquaculture model based on an integrated analysis of liver histology, biochemistry, transcriptomics, and metabolomics data

Meng Yuan[1], Jianfang Guo[1], Yifei Sun[1], Zhihao Liu[1], Yibo Zhao[1], Yikai Li[1], Yongtao Tang[2], Tianxi Fu[3], Chuanjiang Zhou[1,*]

[1] College of Life Sciences, Henan Normal University, No. 46, Jianshe East Road, Muye District, Xinxiang 453007, China; 18888526797@163.com (M.Y.); 19712773098@163.com (J.G.); syf581031@gmail.com (Y.S.); 13849013501@163.com (Z.L.); 13007527278@163.com (Y.Z.); liyikai119@163.com (Y.L.)
[2] College of Fisheries, Henan Normal University, Xinxiang 453007, China; xiaomitang@126.com
[3] Zhejiang Xinxin Tianen Aquatic Feed Co., Ltd., Jiaxing 314006, China; futianxi2004@126.com
* Correspondence: chuanjiang88@163.com; Tel.: +86-373-3326500

This file includes:

A. Supplementary notes

note S1 to S4

B. Supplementary figures

Figure S1 to S2

C. Supplementary tables

Table S1 to S9

D. References

A. Supplementary notes

Note S1 Paraffin Sectioning and H&E Staining

Liver tissues fixed in 4% universal tissue fixative were removed and washed in 70% ethanol on a horizontal shaker until the residual fixative was cleared. The tissues were then dehydrated through a graded ethanol series, followed by clearing in xylene until they became transparent. Subsequently, the samples were infiltrated with molten paraffin in a water bath at appropriate temperatures through multiple steps. The paraffin-infiltrated tissues were embedded in paraffin blocks, cooled to solidify, trimmed, and mounted in embedding cassettes. Serial sections were prepared using a paraffin slicer. The sections were floated on a water bath, mounted onto glass slides, labeled, and dried in a slide dryer. For histological staining, the sections were deparaffinized in xylene and rehydration through a graded ethanol series. The slides were then stained with hematoxylin, differentiated, counterstained, and subsequently stained with eosin. After staining, the sections were rinsed and dehydrated through graded ethanol, cleared in xylene, and mounted with neutral resin. Coverslips were carefully applied to avoid air bubbles, and the slides were air-dried before microscopic observation and image acquisition.

Note S2 Sample Preparation and Sequencing for Transcriptomic Analysis

Total RNA was extracted from liver tissues of the three density groups (LD, MD, and HD) using Trizol reagent according to the manufacturer's instructions, with RNA integrity and concentration assessed prior to library construction. mRNA was enriched using Oligonucleotide(dT) magnetic beads, fragmented, and reverse-transcribed into cDNA, followed by end repair, A-tailing, adapter ligation, fragment selection, PCR amplification, and purification to construct sequencing libraries. Library quality was evaluated using a Qubit fluorometer, qPCR, and an Agilent Bioanalyzer 2100, after which qualified libraries were sequenced on an Illumina platform. Raw reads were processed using fastp software [75] to remove adapters and low-quality reads, yielding clean data for downstream analysis, and quality metrics (Q20, Q30, GC content) were calculated to assess sequencing quality.

Note S3 Sample Preparation and Metabolite Identification for Metabolomics Analysis

A 100 μL aliquot of the sample was mixed with 400 μL of extraction solvent containing an isotopically labeled internal standard (methanol:acetonitrile = 1:1, v/v), vortexed for 30 s, sonicated in an ice-water bath for 10 min, and incubated at −40 °C for 1 h to precipitate proteins. The samples were centrifuged at 12,000 rpm (13,800 × g, radius 8.6 cm) at 4 °C for 15 min, and the supernatant was collected for analysis. Equal volumes of supernatant were pooled to generate quality control (QC) samples.Chromatographic separation was performed using a Vanquish ultra-high-performance liquid chromatography system (Thermo Fisher Scientific) equipped with a Waters ACQUITY UPLC BEH Amide column (2.1 mm × 50 mm, 1.7 μm). The mobile phases consisted of Phase A (aqueous solution containing 25 mmol/L ammonium acetate and 25 mmol/L ammonia) and Phase B (acetonitrile). The autosampler temperature was set at 4 °C, and the injection volume was 2 μL.Mass spectrometry analysis was performed using an Orbitrap Exploris 120 mass spectrometer (Thermo Fisher Scientific) with data acquired in both positive and negative ion modes under Xcalibur software [76] control. The operating

parameters were as follows: sheath gas flow rate, 50 Arb; auxiliary gas flow rate, 15 Arb; capillary temperature, 320 °C; resolution, 60,000 for MS^1 and 15,000 for MS^2; collision energy (SNCE), 20/30/40; and spray voltage, 3.8 kV (positive mode) or −3.4 kV (negative mode). Raw data were converted into mzXML format using ProteoWizard [77]. Metabolite identification was performed using a custom R-based program [78] with BiotreeDB V3.0 as the reference database. Metabolites were annotated using the Lipidmaps database.

Note S4 Integration of β-alanine, histidine, and tyrosine metabolism with immune regulation under high-density stress

However, this redirection comes at a cost. Due to reduced flux of Ser into one-carbon metabolism, insufficient methyl donors are produced for the Met remethylation cycle, leading to significant accumulation of the methyltransferase inhibitor SAH. SAH accumulation implies that nearly all methylation-dependent biological processes—including DNA/RNA methylation, phospholipid synthesis, creatine synthesis, and neurotransmitter metabolism—may be inhibited. This trade-off strategy has also been observed in other fish species: in Nile tilapia (*Oreochromis niloticus*), hepatic phospholipid remodeling—a process highly dependent on methyl supply—was confirmed as a key metabolic adaptation to density stress [67]. Additionally, disruptions in polyamine metabolism reveal another dimension of this cost. In the Arg biosynthetic pathway, upregulation of ODC drives putrescine synthesis, potentially representing a compensatory repair attempt in response to severe tissue damage (nuclear pyknosis and disrupted hepatic cords). However, impaired downstream metabolism leads to accumulation of the cytotoxic substance β-aminopropionaldehyde, while inhibition of the uridine degradation pathway reduces β-alanine production, ultimately diminishing acetyl-CoA supply and fatty acid synthesis. This indicates that, under a strategy prioritizing antioxidant defense, the liver's energy reserve capacity and its ability to clear metabolic waste may be simultaneously declining. This phenomenon aligns with the findings of Zahedi et al [69]: polyamine metabolism disorders are closely associated with tissue damage, and abnormalities in these pathways serve as a key indicator of the transition from compensatory to exhausted physiological function. Finally, the coordinated downregulation of β-alanine metabolism, alongside disruptions in histidine and tyrosine metabolism, may reflect a broader suppression of energy metabolism and immune regulation. The uridine degradation pathway, which serves as a key source of β-alanine, appeared to be suppressed, as suggested by the significant downregulation of *dpydb, dyps*, and *upb1*, may have led to a marked reduction in the intermediate metabolite 5,6-dihydrouracil and decreased β-alanine production, a finding consistent with KEGG pathway annotation for zebrafish (*Danio rerio*) where downregulation of *dpydb* and *upb1* directly reduces β-alanine synthesis (https://www.kegg.jp/entry/dre00410)(8 April 2026).

Concomitant with β-alanine deficiency, the expression levels of key genes such as *abat* and *aldh6a1* are significantly downregulated, further blocking acetyl-CoA production and ultimately leading to marked inhibition of fatty acid biosynthesis. In parallel, the reprogramming of histidine metabolism provides a molecular link to the observed immune response patterns: histidine flux was redirected away from energy production and one-carbon metabolism toward histamine synthesis, while the methylation-dependent inactivation pathway of histamine was impaired due to downregulation of *hnmt* (histamine

N-methyltransferase), leading to an accumulation of N-acetylhistamine—a metabolite formed via an alternative acetylation-dependent inactivation route. This shift suggests that the organism relies on acetylation rather than methylation for histamine clearance under high-density stress, aligning with the biochemical observation that IL-1β levels, which were significantly elevated in the MD group, showed a slight decline in the HD group, indicating a transition from immune activation toward immune suppression. Consistent with this interpretation, a study on high-density stress in two-spot catfish (*Ompok bimaculatus*) reported that pro-inflammatory cytokines such as IL-1β were initially elevated in the kidney and liver but eventually became suppressed under prolonged stress [58], a pattern that parallels the findings of the present study. Collectively, the multi-dimensional metabolic adaptations in the HD group—while initially compensatory—ultimately lead to methyl donor depletion, disrupted polyamine metabolism, impaired energy production, and a shift from immune activation to suppression, driving the liver from a state of compensation toward functional exhaustion. These findings may provide a molecular basis for the observed growth suppression, immune dysregulation, and sporadic mortality in fish subjected to long-term high-density stress under the "168" aquaculture model.

B. Supplementary figures

Figure S1 Diagram of the 168 Aquaculture Model

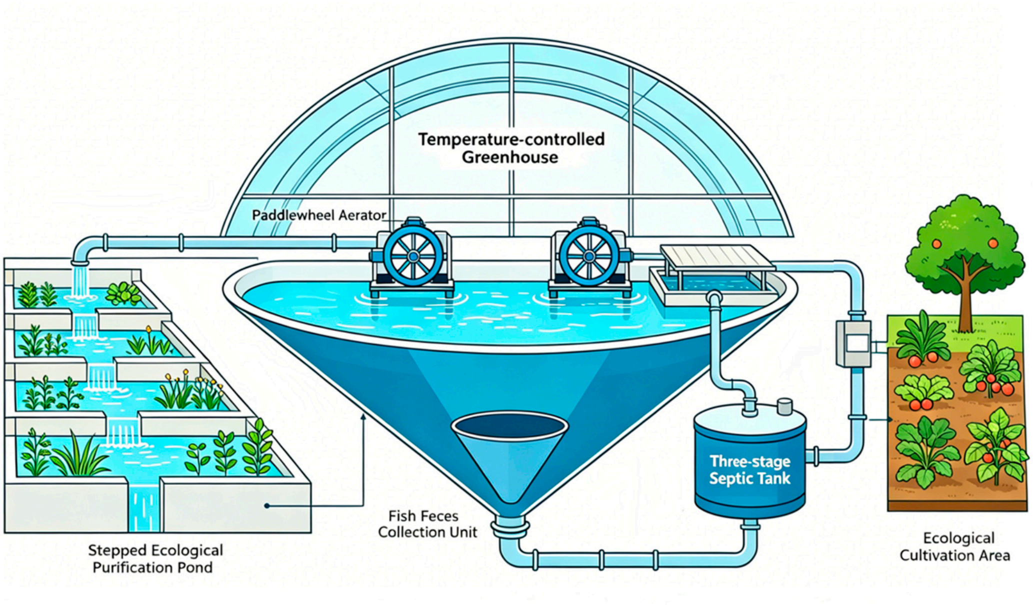

Figure S2 Core regulatory network of DEGs and DEMs in histidine metabolism

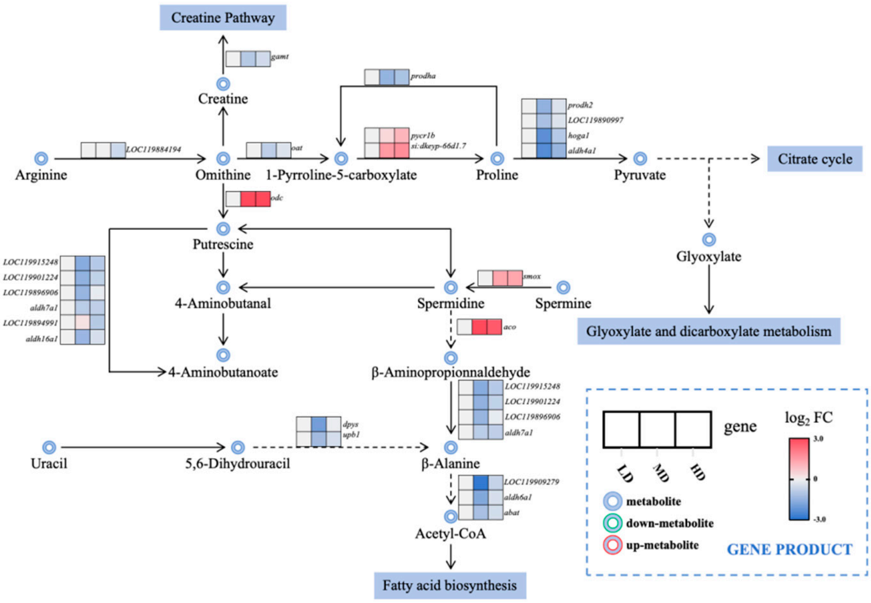

C. Supplementary tables

Table S1 Feeding rates of largemouth bass at the "168" Aquaculture Base

Specification	Body weight range	Feeding rate (% body weight/day)
80-500 fish/kg	1-6.5g	6-8%
40-80 fish/kg	6.5-12.5g	4-6%
10-40 fish/kg	12.5-50g	2.5-4%
—	50-100g	2-2.5%
—	100-200g	1.5-2%
—	200-300g	1-2%
—	300-400g	1-1.5%
—	> 400g	0.5-1%

Table note: Feeding rates are presented as a percentage of body weight per day under optimal water temperature conditions. "—" indicates that the corresponding information was not provided in the original table.

Table S2 Water Quality Indicators 14 Days before Sampling

Date	Density Group	Temperature (℃)	*P*	Ammonia Nitrogen (mg/L)	*P*	Nitrite nitrogen (NO_2^--N)	*P*	Dissolved Oxygen (DO)	*P*	pH	*P*
	LD	25.46±0.38		0.2±0		0.1±0.04		10.44±0.48		7.3 ± 0.4	
2026/3/1	MD	25.58±0.38	ns	0.2±0	ns	0.07±0.03	ns	9.68±1	ns	7.1 ± 0.5	*
	HD	25.3±0		0.2±0		0.09±0.05		9.66±0.79		8.1 ± 0.4	
	LD	25.04±0.58		0.24±0.09		0.11±0.04		8.8±0.77		7.9 ± 0.5	
2026/3/2	MD	25.54±0.43	ns	0.2±0	ns	0.07±0.03	ns	8.2±0.14	ns	7.4 ± 0.4	ns
	HD	25.3±0		0.24±0.09		0.08±0.04		8.8±0.82		7.2 ± 0.4	
	LD	25.1±0.34		0.2±0		0.11±0.04		7.52±0.56		7.6 ± 0.3	
2026/3/3	MD	25.8±0.19	*	0.24±0.09	ns	0.08±0.03	ns	9.32±1.07	*	7.0 ± 0.5	ns
	HD	25.58±0.44		0.2±0		0.11±0.04		9.02±0.83		7.3 ± 0.4	
	LD	25.44±0.38		0.2±0		0.09±0.04		9.9±0.42		7.1 ± 0.5	
2026/3/4	MD	25.78±0.2	ns	0.2±0	ns	0.07±0.03	ns	8.44±0.65	ns	7.5 ± 0.3	ns
	HD	25.56±0.09		0.24±0.09		0.1±0.05		9.26±0.78		7.8 ± 0.6	
	LD	25.34±0.18		0.2±0		0.1±0.04		10.8±0		7.4 ± 0.4	
2026/3/5	MD	25.76±0.22	ns	0.28±0.11	ns	0.07±0.03	ns	9.8±1.01	ns	7.2 ± 0.4	ns
	HD	25.54±0.13		0.2±0		0.07±0.03		10.1±0.35		7.6 ± 0.3	
	LD	25.22±0.08		0.2±0		0.11±0.04		10.25±0.87		8.3 ± 0.3	
2026/3/6	MD	25.64±0.33	*	0.2±0	ns	0.07±0.03	ns	9.4±0.82	ns	7.3 ± 0.4	*
	HD	25.38±0.04		0.2±0		0.09±0.05		9.72±0.27		7.1 ± 0.5	

	LD	25.08±0.19		0.24±0.09		0.11±0.04		10.4±0		7.5 ± 0.3	
2026/3/7	MD	25.54±0.43	ns	0.2±0	ns	0.07±0.03	ns	9.76±1.04	ns	7.9 ± 0.5	ns
	HD	25.22±0.18		0.2±0		0.09±0.05		9.8±0.34		7.1 ± 0.5	
	LD	25.2±0.27		0.2±0		0.11±0.05		9.4±0.36		7.5 ± 0.3	
2026/3/8	MD	25.3±0.51	ns	0.2±0	ns	0.09±0.03	ns	10.1±0.2	ns	7.9 ± 0.6	ns
	HD	24.66±0.42		0.2±0		0.09±0.05		9.92±0.36		7.4 ± 0.4	
	LD	25.025±0.39		0.25±0.1		0.1±0.04		9.88±0.4		7.2 ± 0.7	
2026/3/9	MD	25.2±0.75	ns	0.28±0.11	ns	0.08±0.03	ns	9.4±0.82	ns	8.1 ± 0.4	*
	HD	25.34±0.53		0.24±0.09		0.11±0.07		9.64±0.22		7.0 ± 0.5	
	LD	24.725±0.5		0.25±0.1		0.11±0.03		9.43±0.98		7.3 ± 0.3	
2026/3/10	MD	25.08±0.85	ns	0.2±0	ns	0.08±0.03	ns	9.28±0.81	ns	7.1 ± 0.5	ns
	HD	24.68±0.08		0.2±0		0.13±0.08		9.72±0.53		7.5 ± 0.3	
	LD	24.8±0.38		0.2±0		0.11±0.03		9.8±0.34		7.1 ± 0.5	
2026/3/11	MD	24.98±0.25	ns	0.2±0	ns	0.08±0.03	ns	9.38±0.83	ns	7.4 ± 0.4	ns
	HD	24.98±0.29		0.24±0.09		0.13±0.04		10±0.34		7.2 ± 0.4	
	LD	24.825±0.46		0.2±0		0.13±0.03		8.93±0.4		8.3 ± 0.5	
2026/3/12	MD	25.2±0.17	*	0.2±0	ns	0.14±0.05	ns	9.74±0.74	ns	7.5 ± 0.3	*
	HD	25.6±0.41		0.2±0		0.12±0.03		9.58±0.37		7.1 ± 0.5	
	LD	24.75±0.41		0.2±0		0.13±0.03		8.96±0.86		7.4 ± 0.4	
2026/3/13	MD	25.54±0.51	ns	0.24±0.09	ns	0.08±0.03	ns	9.2±0.76	ns	7.2 ± 0.4	*
	HD	25.72±0.29		0.2±0		0.12±0.04		9.46±0.85		8.3 ± 0.3	
	LD	25±0.39		0.25±0.1		0.13±0.03		10.08±0.72		7.1 ± 0.5	
2026/3/14	MD	25.66±0.33	ns	0.2±0	ns	0.08±0.03	ns	9.12±0.78	ns	7.5 ± 0.3	ns
	HD	25.16±0.36		0.24±0.09		0.14±0.04		9.52±0.29		7.9 ± 0.3	

Table note: In this study, ammonia nitrogen concentration was measured using a portable colorimeter (precision: 0.1 mg/L), and the data were retained to one decimal place according to the instrument resolution. During the monitoring period, the ammonia nitrogen concentration was maintained below the safety threshold of 0.4 mg/L, with 0.2 mg/L serving as a reference value for good water quality in routine management. Therefore, the ammonia nitrogen values in the table are primarily 0.2 and 0.4 mg/L, indicating that the ammonia nitrogen levels remained within a controllable range and did not cause stress to *Micropterus salmoides*. The Kruskal-Wallis H test was performed for group comparisons at each sampling time point, *: $p < 0.05$;, ns: not significant ($p > 0.05$).

Table S3 Measurement Results of Morphological Indicators

Group	Body Weight (kg)	Total Length (cm)
LD	0.15±0.03	20.19±1.23
MD	0.24±0.04	22.70±1.21
HD	0.46±0.06	30.20±0.71

Table S4 Average stocking density of largemouth bass

Group	Mean Body Weight (kg)	Mean Weight Density (kg/m^3)
LD	0.15	2.53125
MD	0.25	4.21875
HD	0.45	7.59375

Table S5 Summary of kit information

Assay	Kit Name	Catalog No.
Protein	Protein Kit	A045-4
CAT	CAT Kit	A007-1-1
MDA	MDA Kit	A003-1-1
GSH-Px	GSH-Px Kit	A005-1-1
Cortisol	Cortisol Kit	YJ32097
GH	GH Kit	YJ32085
IL-1β	IL-1β Kit	YJ32143

Table S6 Sequences of primers used for qRT-PCR in this study

Gene name	Primer sequences (Forward) (5[illegible]3[illegible])	Primer sequences (Reverse) (5[illegible]3[illegible])
gapdh	TGGCAGGTGTCCCAGTTCAG	GCCATGATGCTCCTTTCCAAG
smox	TTCATGCGAGGCTCCTACTC	GCCTTAGTGCTGTTGGCGTA
aco2	GCACTGGAGCCATCGTAGAA	GGTCGTGGCTCCAATCTCTG
mat2ab	CATGCCACAGTGGACTACCA	GGCCACAAGAACATTGCAGG
dpys	TTGTGAATGAGGACTGCTCCG	ATAACAAGCACTCCTGCTGGG
agxtb	CTGGCAGACGCTCTCAAGAA	GCTGCAGTCCAAAGCACATAG
grhprb	GCGGTCTGCTGTGCTTTAAC	CCACCTCGGCTTGTGTTGAT
pycr1b	GGTTGAGCAAAACGGTAGCC	CCCGACAGAGCTCCCTAAGT
glsb	TCGGACCCATGGATTACGAG	CTCAGTGGGGACACCTTTT

Table S7 Transcriptome Sequencing Data Statistics

Samples	Clean reads	Clean bases	GC Content	%≥Q30
LD1	22,761,805	6,828,541,500	48.63%	96.49%
LD2	21,310,893	6,393,267,900	48.35%	96.75%
LD3	27,076,114	8,122,834,200	48.33%	96.60%
MD1	22,829,270	6848,781,000	48.21%	96.74%
MD2	20,825,778	6,247,733,400	47.73%	96.55%
MD3	24,265,629	7,279,688,700	48.41%	96.52%
HD1	25,561,911	7,668,573,300	48.19%	97.00%
HD2	21,790,485	6,537,145,500	48.05%	96.55%
HD3	21,490,514	6,447,154,200	48.18%	96.73%

Table S8 Summary of DEGs

Group	DEG Number	up-regulated	down-regulated
LD vs MD	1962	1040	922
LD vs HD	1315	778	537

Table S9 Functional annotation of LOC genes

Gene ID	Protein Name	GO Molecular Function	KEGG ID	KEGG Pathway
LOC119903997	cystathionine beta-synthase-like	cystathionine beta-synthase activity	K01697	Glycine, serine and threonine metabolism; etc.
LOC119917301	phosphoglycerate mutase 1-like	bisphosphoglycerate mutase activity; phosphoglycerate mutase activity	K01834	Glycolysis / Gluconeogenesis; etc.
LOC119915248	aldehyde dehydrogenase family 9 member A1	oxidoreductase activity, acting on the aldehyde or oxo group of donors, NAD or NADP as acceptor	K00149	Glycolysis / Gluconeogenesis; etc.
LOC119909279	methylmalonate-semialdehyde dehydrogenase [acylating], mitochondrial-like	methylmalonate-semialdehyde dehydrogenase (acylating) activity	K00140	Valine, leucine and isoleucine degradation; etc.

LOC119905685	serine hydroxymethyl-transferase, mitochondrial-like	glycine hydroxymethyl-transferase activity; threonine aldolase activity	K00600	Glycine, serine and threonine metabolism; etc.
LOC119903905	histamine N-methyltransferase-like	N-methyltransferase activity	K00546	Histidine metabolism
LOC119901224	aldehyde dehydrogenase family 9 member A1	oxidoreductase activity, acting on the aldehyde or oxo group of donors, NAD or NADP as acceptor	K00149	Glycolysis / Gluconeogenesis; etc.
LOC119896906	aldehyde dehydrogenase, mitochondrial	oxidoreductase activity, acting on the aldehyde or oxo group of donors, NAD or NADP as acceptor	K00128	Glycolysis / Gluconeogenesis; etc.
LOC119894991	diamine acetyltransferase 1-like	diamine N-acetyltransferase activity	K00657	Arginine and proline metabolism; Ferroptosis
LOC119891844	betaine-homocysteine S-methyltransferase 1-like	betaine-homocysteine S-methyltransferase activity; S-methyltransferase activity	K00544	Glycine, serine and threonine metabolism; Cysteine and methionine metabolism
LOC119891819	D-amino-acid oxidase-like	D-amino-acid oxidase activity; FAD binding	K00273	Glycine, serine and threonine metabolism; etc.
LOC119890997	aspartate aminotransferase, mitochondrial	L-aspartate: 2-oxoglutarate aminotransferase activity; pyridoxal phosphate binding	K14455	Arginine biosynthesis; etc.
LOC119884194	arginase-1	arginase activity; manganese ion binding	K01476	Arginine biosynthesis; etc.
LOC119882202	sarcosine dehydrogenase, mitochondrial	oxidoreductase activity	K00314	Glycine, serine and threonine metabolism
